# CDK4/6 Inhibitors in Combination Therapies: Better in Company Than Alone: A Mini Review

**DOI:** 10.3389/fonc.2022.891580

**Published:** 2022-05-27

**Authors:** Gian Luca Rampioni Vinciguerra, Maura Sonego, Ilenia Segatto, Alessandra Dall’Acqua, Andrea Vecchione, Gustavo Baldassarre, Barbara Belletti

**Affiliations:** ^1^ Division of Molecular Oncology, Centro di Riferimento Oncologico di Aviano (CRO), Istituto di Ricovero e Cura a Carattere Scientifico (IRCCS), National Cancer Institute, Aviano, Italy; ^2^ Department of Cancer Biology and Genetics and Comprehensive Cancer Center, The Ohio State University, Columbus, OH, United States; ^3^ Department of Clinical and Molecular Medicine, Faculty of Medicine and Psychology, Sant’Andrea Hospital, University of Rome “Sapienza”, Rome, Italy

**Keywords:** CDK4/6 inhibitors, drug resistance, small inhibitors, chemotherapy, combination therapy, endocrine therapy

## Abstract

The cyclin D-CDK4/6 complexes play a pivotal role in controlling the cell cycle. Deregulation in cyclin D-CDK4/6 pathway has been described in many types of cancer and it invariably leads to uncontrolled cell proliferation. Many efforts have been made to develop a target therapy able to inhibit CDK4/6 activity. To date, three selective CDK4/6 small inhibitors have been introduced in the clinic for the treatment of hormone positive advanced breast cancer patients, following the impressive results obtained in phase III clinical trials. However, since their approval, clinical evidences have demonstrated that about 30% of breast cancer is intrinsically resistant to CDK4/6 inhibitors and that prolonged treatment eventually leads to acquired resistance in many patients. So, on one hand, clinical and preclinical studies fully support to go beyond breast cancer and expand the use of CDK4/6 inhibitors in other tumor types; on the other hand, the question of primary and secondary resistance has to be taken into account, since it is now very clear that neoplastic cells rapidly develop adaptive strategies under treatment, eventually resulting in disease progression. Resistance mechanisms so far discovered involve both cell-cycle and non-cell-cycle related escape strategies. Full understanding is yet to be achieved but many different pathways that, if targeted, may lead to reversion of the resistant phenotype, have been already elucidated. Here, we aim to summarize the knowledge in this field, focusing on predictive biomarkers, to recognize intrinsically resistant tumors, and therapeutic strategies, to overcome acquired resistance.

## Introduction

The correct progression through the cell cycle is a tightly controlled process that ensures that one cell properly divides into two daughter cells carrying the exact same genetic material.

Cells irreversibly commit to enter the mitotic cell cycle during the G1 phase and this commitment depends on the phosphorylation and degradation of the retinoblastoma protein (Rb) that, in turn, releases the transcription factor E2F1. Phosphorylation of Rb is a key event, tightly regulated by the sequential action of the cyclin-dependent kinase 4 and 6 (CDK4, CDK6) complexed with a positive regulatory D-type cyclin subunit, followed by activation of cyclin E/CDK2 complexes. Extracellular signals and several different molecular pathways regulate the expression of cyclins, CDK and CDK inhibitors, particularly p16^ink4^, p21^Cip1^ and p27^Kip1^. The dysregulation of this circuit often drives the uncontrolled proliferation, observed in many human cancers (1).

Therefore, components of the cell-cycle machinery have been longstanding seen as optimal targets in the field of oncology.

However, initial attempts to block CDK activity in patients were definitely unsuccessful, due to both the low therapeutic index and the high toxicity profiles that these pan-CDK inhibitors displayed, severely lowering the expectations for this type of targeted therapy (2).

The advent of the first CDK4/6 specific inhibitor (i.e. PD0332991, then renamed Palbociclib) has drastically changed this view when preclinical data demonstrated that this compound was able to block cancer cells in G1 phase of the cell cycle, inhibiting CDK4/6-cyclin D complexes with exquisite selectivity and displaying very promising antitumor activities in mice (3–6). Notwithstanding these very promising results obtained in laboratory, when the activity of Palbociclib in combination with anti-estrogen therapy in patients with metastatic hormone receptor positive (HR+) breast cancer (BC) was firstly reported, it was unexpected and impressive, stimulating the rapid design and clinical development of other CDK4/6 inhibitors (CDK-i) (7–9). Currently, three selective CDK-i are FDA-approved for the treatment of HR+ advanced BC: Palbociclib, Ribociclib and Abemaciclib, and active clinical trials are ongoing to test their efficacy in other settings of BC, as well as in other neoplasms, based on the encouraging results obtained in a wide spectrum of preclinical settings (2, 10, 11).

However, now that a long enough follow-up of the patients is available, clinical data reveal that about 30% of patients with advanced stage BC do not respond to CDK-i and a very large proportion of the patients eventually acquire resistance. *In vitro*, neoplastic cells that acquire CDK-i resistance become more aggressive, displaying a distinct genomic, transcriptomic and proteomic profile that results in the upregulation of epithelial-mesenchymal transition (EMT), appearance of stem-like features, increased migratory and invasive capacity (12, 13).

To date, many studies have contributed to clarify how CDK-i exert their anti-tumor effect and, also, the molecular mechanisms governing CDK-i resistance, in the attempt to identify predictive biomarkers of response and pharmacological strategies to overcome it (14). From these studies, we have learned that CDK-i molecules are quite dissimilar from each other, differing for their affinity to CDK4 and CDK6 (i.e. Ribociclib and Abemaciclib are more active against CDK4, Palbociclib has similar activity against both kinases), pharmacokinetics and spectrum of toxicity (14). Despite these differences, acquired resistance to one CDK-i confers cross-resistance to the others (15), at least if we consider their canonical function of controlling CDK4/6-Rb-E2F axis.

It is known that CDK-i do not trigger apoptosis, but significantly induce cell cycle arrest and senescence in Rb-proficient cells (16). However, several studies in HR+ breast and lung cancer models indicate that Palbociclib interacts also with lipid kinases, suggesting that it may affect multiple signaling pathways, including the one of PI3K/AKT/mTOR, even if their inhibition, at least *in vitro*, is weak (17, 18). Moreover, Palbociclib indirectly stabilizes and activates the proteasome, *via* reduction of ECM29, a protein that disassembles the proteasome contributing to the induction of a senescent phenotype (18). In bladder cancer models, Palbociclib impinges on FOXM1 phosphorylation and activation, exerting its anti-tumor activity independently from Rb-status (19). Also, Abemaciclib has been reported to interact with the transporters ATP-binding cassettes ABCB1 and ABCG2, which play a pivotal role in chemo-resistance, pumping drugs out of cancer cells. In this context, Abemaciclib is able to inhibit ABCB1 and ABCG2-mediated drug efflux resulting in an increased intracellular concentration of therapeutic compounds that could revert the phenotype of tumors with multidrug resistance (20).

Even if the significance of this non-canonical functions will need to be assessed in the clinical setting, these studies clearly indicate that CDK-i activity is not limited to lowering Rb phosphorylation and blocking the cell cycle, but it involves pathways that could be exploited by resistant tumors to overcome CDK4/6 inhibition (20).

Aim of this review is to recapitulate the recent insights into the interaction between CDK-i and other antitumor agents, the mechanisms of CDK-i resistance, and the upcoming approaches to overcome it. As summarized in **Table 1**, neoplastic cells may adopt different strategies to overcome pharmacological inhibition of CDK4/6, involving both cell cycle specific and nonspecific mechanisms, eventually resulting in phosphorylation and re-activation of Rb (68). Cell cycle-specific mechanisms encompass the incomplete inactivation of CDK4/6, the capability of cyclin E1/E2-CDK2 complex to initiate the phosphorylation of Rb and the direct inactivation of Rb (69), while cell cycle-nonspecific mechanisms rely on different pathways, especially like Receptor Tyrosine Kinases (RTK) and PI3K-AKT-mTOR axis (68). As discussed below in more detail, CDK-i resistance could also be the result of *de novo* mutations, such as the inactivation of Rb (70), the amplification of cyclin E1 (CCNE1) (23), CDK4 (25) or CDK6 (27).

**Table 1:** Table summarizes the combination therapies comprising a CDK4/6-inhibitor *plus* another compound, tested in either clinical trials or preclinical models, described in the text.

CDK-inhibitor	Combined with		Clinical Trial or Preclinical Moldel	Significance	Reference
	category	compound			
Palbociclib	aromatase inhibitor	Letrozole	PALOMA-2 (NCT01740427)	longer PFS respect to Letrozole alone	(7)
Palbociclib	SERD	Fulvestrant	PALOMA-3 (NCT01942135)	longer OS respect to Fulvestrant alone (not significant)	(47)
Ribociclib	aromatase inhibitor	Letrozole	MONALEESA-2 (NCT01958021)	longer PFS respect to Letrozole alone	(8)
Ribociclib	SERD	Fulvestrant	MONALEESA-3 (NCT02422615)	longer OS respect to Fulvestrant alone	(48)
Ribociclib	goserelin + aromatase inhibititor or Tamoxifen		MONALEESA-7 (NCT02278120)	longer OS respect to endocrine therapy alone	(49)
Abemaciclib	SERD	Fulvestrant	MONARCH-2 (NCT02107703)	longer OS respect to Fulvestrant alone	(36)
Abemaciclib	aromatase inhibitor	Anastrozole or Letrozole	MONARCH-3 (NCT02246621)	longer PFS respect to aromatase inhibitor alone	(9)
Palbociclib	platinum compound	Carboplatin	ovarian cancer	DDR inhibition	(50)
Ribociclib	platinum compound	Cisplatin	ovarian cancer	DDR inhibition	(51)
Palbociclib	taxane	Taxol	pancreatic cancer	DNA-repair inhibition	(52)
Palbociclib	Wee-1 inhibitor	Adavosertib	sarcoma	mitotic catastrophe and senescence induction	(53)
Palbociclib	Wee-1 inhibitor	Adavosertib	HR+ breast cancer	apoptosis of G2 checkpoint dependent cells	(54)
Palbociclib	STAT3-inhibitor	Napabucasin	HR+ breast cancer	blockage of IL6/STAT3-induced resistance	(13)
Palbociclib	PRMT5-inhibitor	Pemrametostat	melanoma	restore of p53 activity	(55)
Palbociclib	MDM2-inhibitor	Nutlin-3	melanoma	restore p53 and p21 expression	(56)
Palbociclib	Src-inhibitor	Saracatinib	colorectal cancer	reduce inhibitory phosphorylation of p27	(57)
CDK6-silencing	Raf-inhibitor	Sorafenib	TN breast cancer	synthetic lethality due to synergistic interaction	(58)
Palbociclib, Ribociclib	MEK-inhibitor	Trametinib, U0126	prostate cancer	revert MAPK-induced resistance to CDK-i	(59)
Ribociclib	PI3K-inhibitor	Alpelisib	TN breast cancer	reduce mTOR activity, increase anti-tumor T-cell response and immunotherapy sensitivity	(60)
Palbociclib	PI3K-inhibitor	Pictilisib (GDC-0941)	HR+ breast cancer	delay insurgence of CDK-i resistance	(23)
Palbociclib	mTOR-inhibitor	Vistusertib	HR+ breast cancer	delay insurgence of CDK-i resistance	(22)
Palbociclib	mTOR-inhibitor	Everolimus	glioblastoma	impact on cancer cell metabolism and improve CDK-i cytotoxicity	(24)
Abemaciclib	mTOR-inhibitor	Everolimus	HR+ breast cancer	inhibit cell growth of CDK-i resistant cells	(21)
Palbociclib	FGFR-inhibitor	FIIN-2, FIIN-3	HR+ breast cancer	counteract FGFR-induced resistance	(61)
Palbociclib	pan-ERBB inhibitor	Afatinib	esophageal carcinoma	synthetic lethality due to synergistic interaction	(62)
Ribociclib	ALK-inhibitor	Ceritinib	neuroblastoma	synthetic lethality due to synergistic interaction	(63)
Palbociclib	Immunotherapy	anti-PD1-mAb	colon cancer model	synergistic interaction	(64)
Abemaciclib	Immunotherapy	anti-PDL1-Ab	mouse models	synergistic interaction	(65)
Palbociclib	autophagy-inhibitor	Hydroxychloroquine	HR+ breast cancer	synergistic interaction	(16)
Ribociclib	YAP-inhibitor	CA3 (CIL56)	esophageal carcinoma	induction of radiation sensitivity	(66)
Abemaciclib	YAP-inhibitor	Verteporfin	pancreatic cancer	synergistic interaction	(67)

CDK-i, CDK4/6-inhibitors; DDR, DNA-damage response; HR, hormone receptor; mAb, monoclonal antibody; OS, overall survival; PFS, progression-free survival; SERD, selective estrogen receptor degrader; TN, triple-negative.

## CDK-i and Endocrine Therapy

Cyclin D–CDK4/6 complexes are hyperactivated and drive uncontrolled tumor proliferation in many cancer types and many preclinical studies demonstrated that, while they are relevant for the growth of many tumor types, they often become essential in breast cancer (BC) (26, 28, 71, 72).

BC is not a single disease, but rather a collection of mammary pathologies heterogeneous in terms of histology, genetic and genomic variations, therapeutic response and clinical outcome. Multiple classifications have been proposed to a better stratification of BC patients, in the attempt to understand the intricate biological mechanisms driving these tumors and to enable more effective clinical trials and treatments. Thanks to gene expression profiling studies (29), surrogate intrinsic subtypes have been established and are typically used in the clinical routine, based on the expression of few key proteins: estrogen receptor (ER), progesterone receptor (PR), human epidermal growth factor receptor 2 (HER2) and the proliferation marker Ki67. Tumors expressing ER and/or PR are termed hormone receptor positive (HR+) or luminal BC, accounting for over 70% of all BC; tumors expressing HER2 are called HER2-enriched BC, accounting for 10-20% of BC; and tumors that do not express ER, PR and HER2 are called triple negative (TN) BC, accounting for 10-15% of BC (30, 73). When CDK4/6i were developed and tested in preclinical studies, cell lines and xenografts representing the luminal BC subtype were shown to be most susceptible to proliferation arrest and tumor shrinkage (31, 74).

Accordingly, when CDK4/6i (Palbociclib, Abemaciclib and Ribociclib) were introduced to the clinic they represented a real breakthrough for treatment of HR+/HER2- luminal BC patients. The addition of CDK4/6i to the standard endocrine therapy showed impressive results, extending median progression free survival and prolonging median overall survival of advanced/metastatic luminal BC patients. Thus, in 2016, PALOMA trials led to the FDA approval of Palbociclib in combination with Letrozole (PALOMA-1, -2, -4) or Fulvestrant (PALOMA-3). In 2017, MONALEESA and MONARCH trials opened the way to the use of Ribociclib and Abemaciclib, in the same clinical setting (36, 47–49). The benefit of CDK4/6i in combination with standard endocrine therapy remains unfortunately still controversial for early stage BC patients and is being investigated in ongoing clinical trials (PALLAS, PENELOPE-B, EarLEE-1, MonarchE) (75).

However, although CDK4/6i offered an improvement in disease control in luminal BC patients, not all women respond to these drugs and many of them develop a secondary resistance.

**Figure 1:**
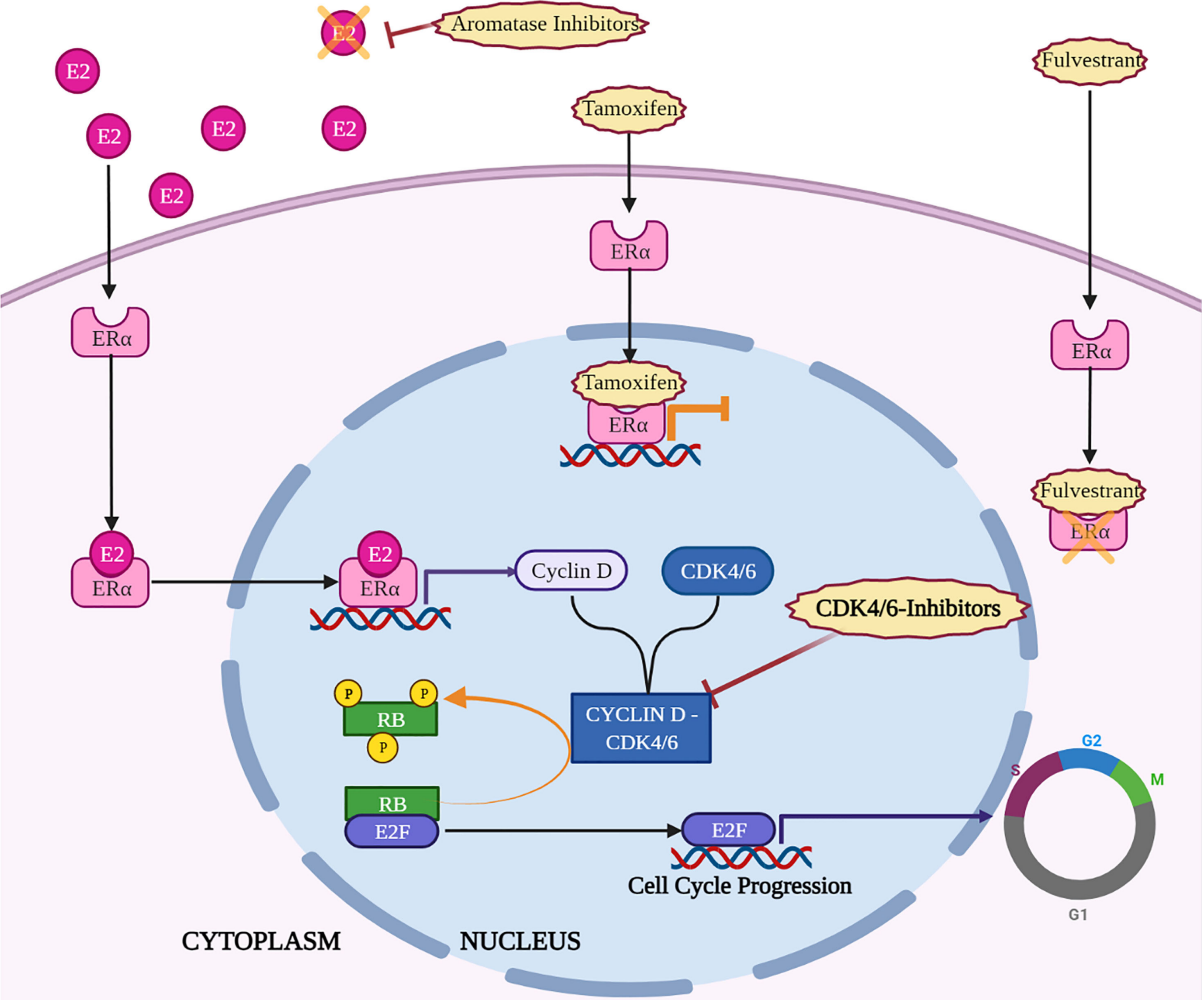
Key mechanisms of action of endocrine therapy and CDK4/6-i in HR+ breast cancer. Created with BioRender.com.

So, many efforts have been made to identify mechanisms underlying CDK4/6i resistance and to understand if resistance to endocrine-therapy could also affect sensitivity to CDK-i. Evidence collected in *in vitro* settings suggests that Palbociclib resistant cells are cross-resistant to endocrine therapy (13), due to the fact that CDK inhibition acts directly downstream of endocrine therapy (20) (**Figure 1**). However, this is not so straightforward in clinical settings: patients with BC harboring mutations in estrogen receptor maintain a general sensitivity to CDK-i (76). In some cases, treatment with CDK-i results even more effective, possibly taking advantage from the same mechanism that leads to endocrine-therapy resistance. For instance, the dysregulation of the mismatch repair complex in BC abrogates the suppression of CDK4 induced by endocrine therapy *via* ATM/CHK2. Thus, the resulting activation of CDK4 determines a resistance to endocrine-therapy but, concomitantly, a higher sensitivity to CDK-i (77). In an opposite way, ectopic overexpression of cyclin E1 or E2 reduced the sensitivity to CDK-i (78) and, in particular, high expression of cyclin E2, was also linked to the tamoxifen-resistant phenotype in HR+ BC cell lines. Interestingly, PALOMA-3 patients with high cyclin E mRNA levels evaluated in metastatic tissue correlated with lower efficacy of the combination of Palbociclib with Fulvestrant (79). Furthermore, CCNE1 gene was found amplified in ctDNA from HR+ patients who do not benefit from the addition of Palbociclib to hormonal therapy (80).

Altogether, combined endocrine and CDK4/6i therapy has certainly changed the course of advanced luminal BC, but new strategies to overcome resistance and reduce recurrence and mortality of these patients are urgently needed.

## CDK-i and Conventional Chemotherapy

Combination of CDK-i with conventional chemotherapeutic drugs has been long debated, due to the fact that cell cycle arrest induced by CDK-i seems in clear conflict with the aim of conventional chemotherapy, which is the targeting of proliferating cells (81).

First evidences accumulated in this field seemed to support this incompatibility. A study conducted in a genetically-engineered mouse model of BC reported that CDK-i impinged on carboplatin efficacy in Rb-proficient tumors (32).

A drug screening performed in pancreatic cancer cell lines, comprising more than 300 anti-cancer compounds combined with Palbociclib, revealed that CDK4/6 inhibition protected cancer cells from chemotherapeutic drugs that targeted mitotic machinery and, thus, require an active cell cycle progression for their action (82). As a consequence, Palbociclib antagonized effects of anti-mitotic agents, such as docetaxel and paclitaxel, the anti-metabolite Gemcitabine and PLK1-inhibitors, a kinase that plays an essential role during mitosis (82). In the same direction, a study conducted on triple negative BC cell lines, showed that administration of Palbociclib together with the genotoxic agent doxorubicin (anthracycline) mitigated the efficacy of the chemotherapy, protecting Rb-proficient cells from the cytotoxic effects of doxorubicin (83).

Altogether, these works strongly indicate that a careful evaluation of the combination treatment, in a schedule-specific and context-specific manner, is needed, in order to avoid possible unwanted interactions between drugs that may eventually lower, instead of heighten, the clinical benefit for the patients.

However, recent studies have partially subverted previous conclusions, demonstrating that the combination between conventional cytotoxic agents and CDK-i can be feasible and effective if administered in the appropriate sequence of time. In this context, our group was among the first demonstrating that sequential administration of carboplatin followed by Palbociclib resulted in synergistic ovarian cancer cell killing, while the same was not true when Palbociclib was administered together or before carboplatin (50). Similarly, administration of CDK-i after antimitotic agents, like taxanes, prevented cellular recovery in different models of pancreatic cancer (52). At mechanistic level, when CDK-i was administered after taxanes, not only it did not interfere with mitotic entry, but it also prevented the expression of PARP induced by E2F, leading to a repression of DNA-repair machinery and a persisting DNA damage, even after taxane suspension (52).

From a totally different perspective, these evidences also led to hypothesize an off-label use of CDK-i to prevent cytotoxicity induced by chemotherapy in highly proliferating normal tissues that harbor an intact Rb pathway. Hematopoietic progenitor cells are highly sensitive to genotoxic agents and their exhaustion (myelosuppression) represents a major adverse effect of many conventional therapeutics, such as ionizing radiation and 5-Fluorouracil. By lengthening the G1-phase of the cell cycle, administration of CDK-i mitigates the toxicity induced by DNA-damage and protects hematopoietic cells (34, 84). This use of CDK-i is largely supported by data in literature and may represent another possibility to combine CDK-i with other drugs (32, 69).

## CDK-i Resistance: Targeting the DNA Damage Response

As previously discussed, some conventional chemotherapeutics specifically work as genotoxic agents and their efficacy is refrained by the capability of neoplastic cells to cope with DNA-damage by activating the DNA-damage response (DDR). DDR pathway is driven by the activity of the protein kinases ATM and ATR, which target checkpoint kinases (Chk1 and Chk2), acting to reduce CDK activity and delaying the cell cycle progression. The arrest of cell cycle eventually creates an extended time window that allows neoplastic cells to recruit DNA-repair-proteins.

Many works have recently highlighted the crosstalk existing between CDK-i and the DDR pathway.

**Figure 2:**
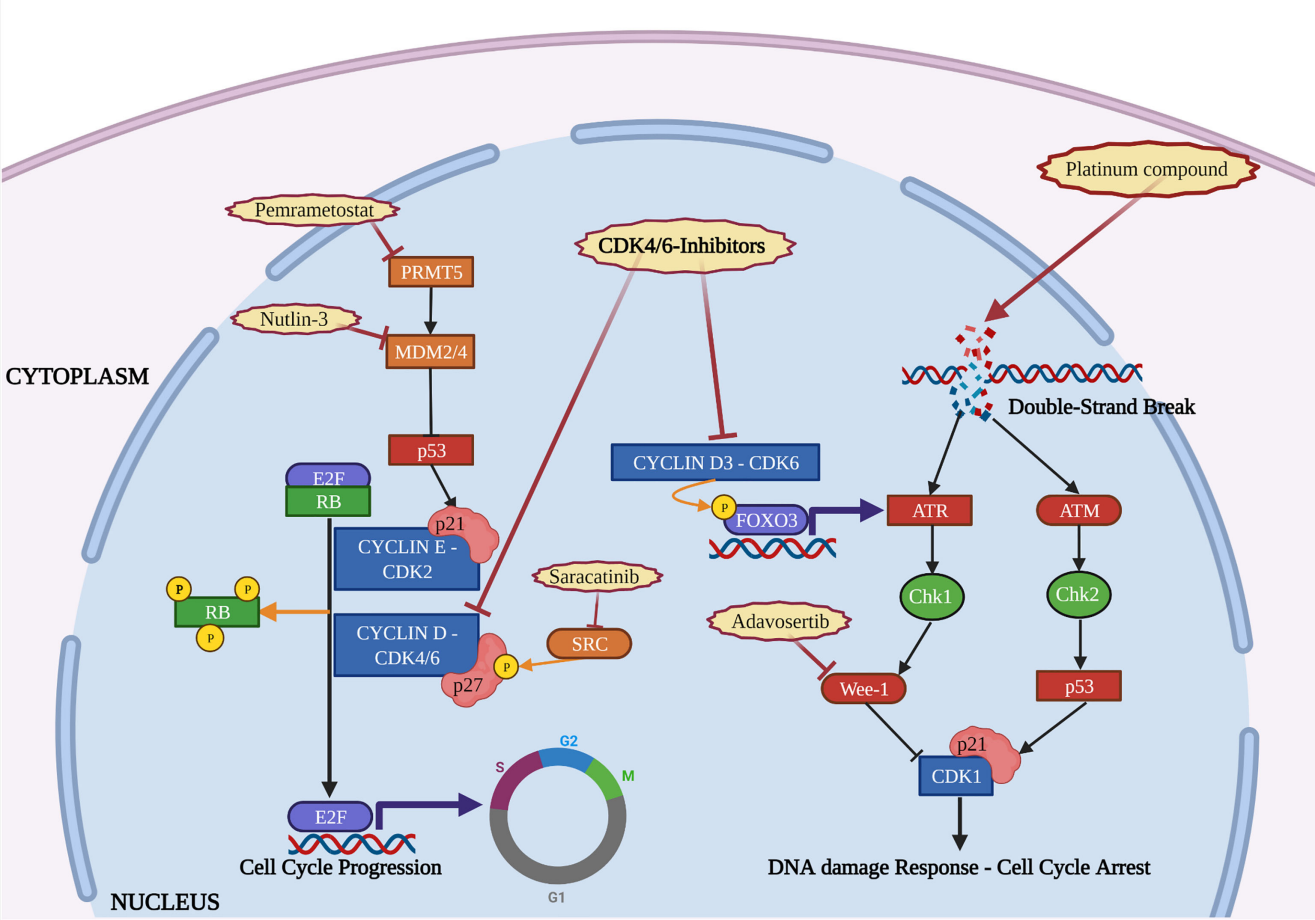
The figure depicts the interactions occurring in the cell nucleus between the activity of CDK4/6-i, DNA damage repair and other molecular pathways. Mechanisms of resistance to CDK4/6-i (orange) and therapeutic strategies to overcome them (red) are reported. Created with BioRender.com.

Platinum exposure induces DNA single and double strand breaks that, in turn, elicits DDR pathway activation. In epithelial ovarian cancer, our group has recently demonstrated that CyclinD3-CDK6 complex stabilizes the transcription factor FOXO3 and promotes transcription of ATR, contributing to DDR activation and, eventually, to cell survival. Significantly, CDK6 expression was higher in recurrent tumor collected from patients who had received platinum-based therapy. CDK6 inhibition counteracted the increased expression of ATR induced by platinum and combination of platinum with Palbociclib resulted in synthetic lethality (50) (**Figure 2**). This observation was confirmed in another work on ovarian cancer, demonstrating that concurrent administration of Ribociclib with cisplatin, followed by maintenance with Ribociclib, was strongly effective in arresting cell growth, preventing Chk-1 activation (51).

Other evidences highlighted the efficacy of CDK-i in combination with inhibitors of Wee-1, a tyrosine kinase that exerts a regulatory role on the timing of mitosis, by inhibiting CDK1 and allowing time for DNA repair (53). Interestingly, in Rb-proficient sarcoma cells, the G1 arrest and release induced by intermittent administration of Palbociclib made cells more sensitive to agents that exert their activity during S-G2 phase. In this context, Wee1-inhibitor Adavosertib (AZD1775) improved efficacy of Palbociclib when administered in a sequential combination treatment (53). The same was observed in HR+ BC cells cross-resistant to both CDK-i and endocrine-therapy, in which administration of Wee1-inhibitor was effective in inducing cell apoptosis due to a strict dependency of these cells from the G2 checkpoint and from repairing DNA damage (54) (**Figure 2**).

Another study identified IL6/STAT3 pathway and DDR deficiency as common tracts among Palbociclib resistant BC cell lines (13). Induction of IL-6 led to phosphorylation of STAT3 *via* JAK, resulting in downregulation of ER, occurrence of EMT and cancer stem-like phenotype that could be counteracted by Napabucasin, a newly developed small inhibitor of STAT3. On the other hand, DDR deficiency sensitized CDK-i resistant cells to either PARP-inhibitors, olaparib and niraparib, or, again, to the Wee1-inhibitor Adavosertib (13).

Another interesting interaction between CDK-i and DDR pathway was shown in melanoma cells, *via* p53. In TP53 wild-type cells, p53 activation contributes to the DDR by inducing the expression of p21^cip1^ (hereafter p21). Suppression of protein arginine methyltransferase 5 (PRMT5) activity by CDK-i represented a key step for the efficacy of these drugs, leading to p53 activation and induction of p21 (55). PRMT5 is an epigenetic modifier that regulates gene expression through methylating arginine residues of histones and non-histone proteins, like several spliceosomal proteins that, in turn, regulate pre-mRNA splicing of the p53-inhibitor MDM4. The inhibition of PRMT5 induced by Palbociclib resulted in pre-mRNA splicing of MDM4, a decreased expression of MDM4 protein and a consequent activation of p53. In melanoma CDK-i resistant cells, Palbociclib failed to decrease MDM4 *via* PRMT5 and, in turn, p53 remained inactive. Administration of PRMT5 inhibitor (Pemrametostat) enhanced the efficacy of Palbociclib in resistant cells and delayed the insurgence of CDK-i resistant phenotype in naïve cells (55).

These evidences were partially confirmed in melanoma patient-derived xenograft (PDX) model, where administration of Nutlin-3, an antagonists of the p53-inhibitor MDM2, was effective in stabilizing p53, restoring p21 expression and counteracting resistance to CDK-i (56, 85). These findings provide the mechanism through which melanomas harboring mutation of TP53 appeared intrinsically resistant to CDK-i. In this model, p21 was sequestered by cyclinD1 overexpression, thereby abrogating its inhibitory activity on CDK2 (56). As discussed below, among the cell cycle-specific mechanisms of CDK-i resistance, hyperactivation of CDK2 is a major one, leading to Rb phosphorylation and cell cycle entry.

## CDK-i Resistance: Targeting the Phosphorylation of p27^kip1^

In literature, one of the first mechanisms reported for CDK-i resistance is the reactivation of CDK2 following down-regulation of p27^kip1^ (hereafter p27), in acute myeloid leukemia (33). Since then, many studies investigating CDK-i resistance have reported the central role of p27, a member of the CIP/KIP family of cyclin-dependent kinase inhibitors, physiologically involved in the regulation of both CDK2 and CDK4/6 complexes (37). p27 is promptly and efficiently downregulated, mainly by degradation, upon mitogenic stimulation. One of the pathways that leads to p27 downregulation is the phosphorylation on its tyrosines 74 and 88 by Src family members. Phospho-p27 has a shorter half-life compared to the unphosphorylated one, reducing its inhibitory activity on cyclin-CDK2 complex (35, 39). Moreover, when phospho-p27 is associated with cyclin D1-CDK4, the resulting complex retains the capability to phosphorylate Rb and is not recognized by CDK-i (38). Intriguingly, in breast cancer biopsies, the immunohistochemical expression of both total p27 and its pY88 form stratified the tumor sensitivity to Palbociclib, supporting the correlation between pY88-p27, CDK4 activity and Palbociclib sensitivity (86).

Taking into account these evidences, blocking the phosphorylation of p27 may represent a valuable strategy to overcome Palbociclib resistance. An extensive screening revealed that, in BC cell lines, Breast tumor-related kinase (Brk) bound p27 with higher affinity than other kinases of the Src family and modulation of Brk affected p27 phosphorylation and Palbociclib sensitivity (87). Furthermore, BC cell lines that expressed an ALTernatively-spliced form of Brk (ALT), lacking the SH1 kinase domain, failed to phosphorylate p27 on its tyrosine residues, resulting in increased CDK4 and CDK2 inhibition following Palbociclib administration (88). Finally, a recent study from our group demonstrated that high levels of phosphorylated Y88 p27 led to increased resistance to Palbociclib, in KRAS-mutated colorectal cancer, but administration of the Src-inhibitor Saracatinib was able to restore Palbociclib sensitivity, both *in vitro* and *in vivo* (57) (**Figure 2**). Interestingly, our study highlighted that regulation of Palbociclib sensitivity by p27 was dependent from the presence of KRAS mutation. This finding is particularly interesting if we consider that upregulation of the Ras pathway represents one of the main cell cycle-nonspecific mechanism of CDK-i resistance (see below).

## CDK-i Resistance: Targeting the Ras-MEK-ERK Axis

**Figure 3:**
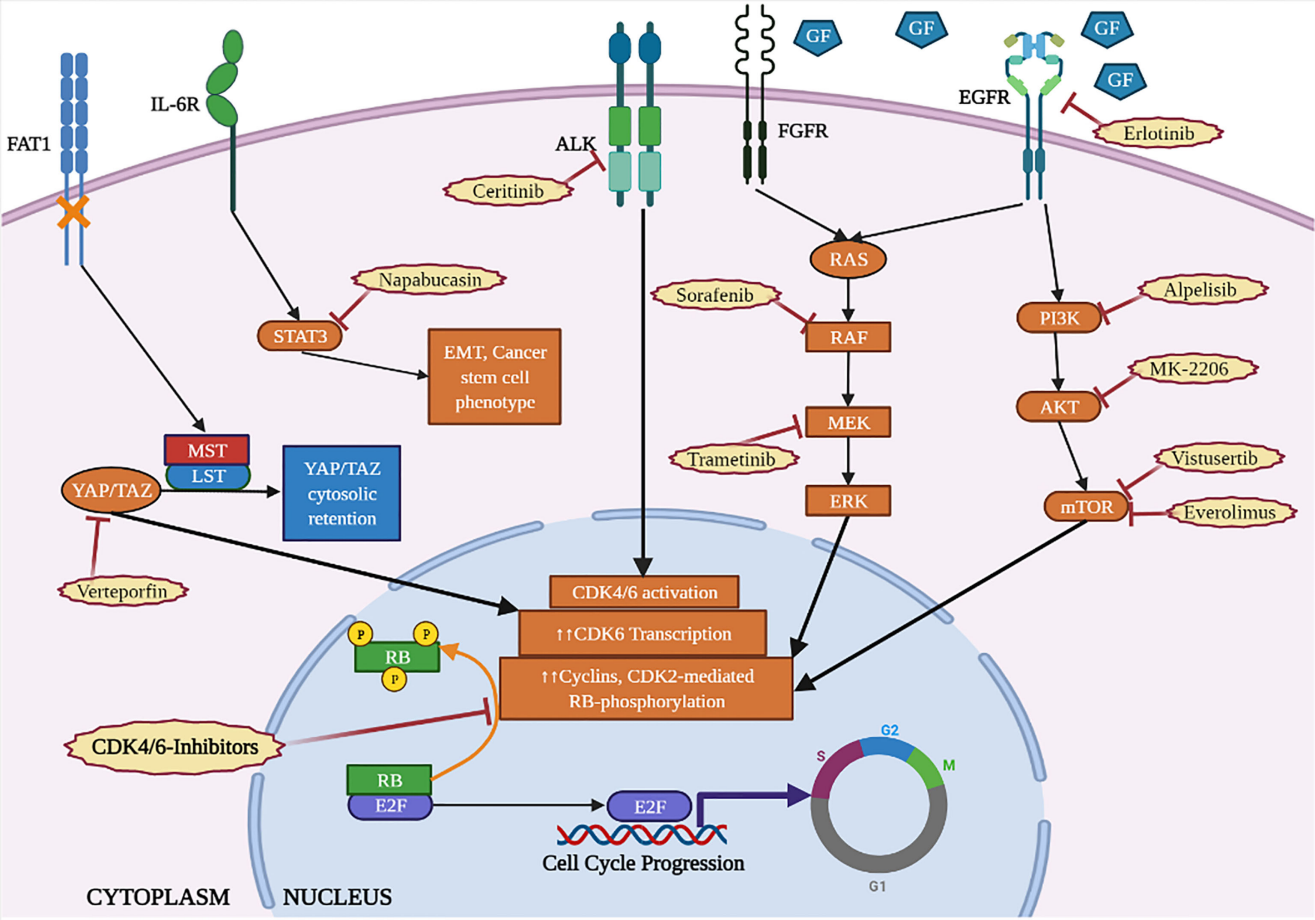
Cytoplasmic cascades involved in the resistance to CDK4/6-inhibitors (orange) and targeted therapies to counteract them (red). Created with BioRender.com.

The Ras-MEK-ERK axis is a signal transduction pathway that acts downstream of several receptors, contributing to the transcriptional and functional regulation of D-type cyclins and CDK4/6. Aberrant activation of Ras pathway plays a crucial role in malignant transformation, in a large number of human tumors. Recently, clinical evidences have highlighted the possible involvement of Ras proteins in inducing resistance to CDK-i (**Figure 3**). In fact, the analysis of circulating tumor DNA collected from patients with BC at baseline and after treatment with CDK-i and endocrine-therapy revealed acquired mutations of different oncogenic drivers and, among others, of RAS (40).

Whole exome sequencing of tumors from BC patients treated with CDK-i in combination with endocrine-therapy (Fulvestrant), revealed that about 10% of CDK-i resistant cases harbored activating mutation of RAS. More importantly, RAS mutations were absent in CDKi-sensitive tumors (89).

RNAseq data from prostate cancer cellular models identified KRAS and RAF overexpression as a hallmark of CDK-i resistance and, accordingly, mass spectrometry profiling identified an enrichment in MAPK activation supported by increased expression of EGF and paracrine activation of EGFR. Noteworthy, the acquired resistance described in this model reflected a kinome rewiring that bypassed CDK-inhibition, without involvement of genomic alterations. As a consequence, CDK-i resistant cells appeared more reliant on MAPK signaling and, consequently, more sensitive to combination treatment with MEK inhibitors (59).

In KRAS-mutated colorectal cancer, combination of MEK and CDK4/6 inhibitors provided a synergistic antitumor activity, both *in vitro* and in PDX models. At mechanistic level, tumor growth impairment was coupled with a decrease in cyclin B1, Rb, Foxm1, Plk1 and phosphorylation of the ribosomal protein S6, a protein phosphorylated by the mTOR pathway and tightly associated with cell cycle progression (90).

Interestingly, a combined genome-scale ORF overexpression screen and a CRISPR knockout screen, performed on melanoma cell lines dependent from NRAS mutation, revealed that the co-occurrence of KRAS mutation drives the resistance to Palbociclib, either when administered as a single agent or in combination with the MEK inhibitor Trametinib (41). This study also highlights that combined inhibition of CDK4/6 and MEK may elicit increased expression or mutation of EGFR-PI3K-AKT-mTOR signaling cascade members, eventually resulting in restored levels of cyclins and/or S6 phosphorylation (41).

Finally, it was also reported that, in Rb-proficient TNBC cells, combination of CDK-i and Raf inhibitor Sorafenib was an effective strategy to induce synthetic lethality (58).

## CDK-i Resistance: Targeting the PI3K-Akt-mTOR Pathway

The serine/threonine kinase mTOR integrates a wide variety of cellular stimuli, including mitogen and nutrient signals to control cell proliferation, cell cycle, cell size and autophagy. One of the main activators of mTOR is the PI3K/AKT axis that forms, together with mTOR, a pathway frequently hyperactivated in cancer and also involved in CDK-i resistance (22) (**Figure 3**).

In preclinical models of BC, CDK-i resistant cells showed a decreased ER expression and a reduced efficacy of anti-estrogen drugs, but a preserved sensitivity to the PI3K-inhibitor Alpelisib and the mTOR-inhibitor Everolimus (21). A synergistic effect of Alpelisib in combination with Ribociclib was observed in Rb-proficient TNBC cell lines, in which PI3K inhibition reduced mTOR activity, inducing cell-cycle arrest and apoptosis (60). Moreover, in a syngeneic murine model of TNBC, combined inhibition of both PI3K and CDK4/6 induced increased activation of tumor-infiltrating T-cells, also suggesting an immunogenic effect, as discussed below (60).

A sensitization screening, aimed at identifying compounds that synergize with Palbociclib in HR+ BC cell lines, revealed that several cytotoxic chemotherapy drugs (paclitaxel, camptothecin, vinorelbine, etc) showed an antagonistic interaction, while PI3K- (GDC-0941), AKT- (MK2206), mTOR- (Everolimus) and IGF1R-inhibitors displayed a synergistic effect (23). Particularly, this study identified two different phases of CDK-i resistance: an early phase, in which cyclin D1 directly interacted with CDK2 instead of CDK4/6, determining a downstream expression of cyclin E2; and a late phase, in which overexpression of cyclin E1 or loss of Rb expression led to cell cycle entry, regardless of the inhibition of cyclin D1-CDK4/6. Importantly, PI3K-inhibition was effective in the early “adaptive phase” of CDK-i resistance, not only impinging on cyclin D1 expression and inducing cell apoptosis, but also delaying the insurgence of the late phase of resistance (23).

In pancreatic cancer cells, it was observed that Palbociclib induced an increase in cyclin D1and E1 levels and resistance was efficiently counteracted by both MEK and mTOR inhibitors (82).

*In vitro*, combined administration of mTOR inhibitor Vistusertib (AZD2014) and Palbociclib induced a durable growth arrest, but not apoptosis, and a delay in the onset of resistance in HR+ BC cells. Vistusertib was also able to induce a reduction of Rb phosphorylation in CDK-i resistant cells, suggesting a possible efficacy in both Palbociclib sensitive and resistant cells (22).

Also in glioblastoma, Palbociclib administration induced an early suppression of downstream mediators of mTOR, like S6, and, as rebound effect, an increased mTOR activity (24). Therefore, the addition of Everolimus to Palbociclib increased benefits against glioblastoma at multiple levels, 1) favoring brain delivery of Palbociclib by impingement on the activity of transporters deputed to the efflux from the blood brain barrier, 2) increasing cytostatic to cytotoxic conversion and 3) blockade of cellular metabolism (24).

## CDK-i Resistance: Targeting the Receptor Tyrosine Kinase (RTK) Pathways

The activation of receptor tyrosine kinases, acting upstream of both Ras and mTOR pathways, represents a crucial signal for neoplastic cell growth, also involved in the resistance to CDK-i. In Palbociclib resistant lung cancer cells, an increased activation of several receptor kinases was observed, such as epidermal growth factor receptor (EGFR), ephrin type-A receptor 1/2 (EphA1/2) and fibroblast growth factor receptor (FGFR) (42). Compared to the naïve cells, Palbociclib resistant cells maintained the same sensitivity to EGFR inhibition, but became significantly more sensitive to FGFR inhibition (42).

Clinical evidences have highlighted the role of FGFR family members in the progression of HR+ BC and in determining the cross-resistance to endocrine therapy and CDK-i (43). Approximately 15% of HR+ BC harbors a FGFR1 amplification that sustains ER pathway activation, even in presence of endocrine therapy (91). A genomic profiling conducted on HR+ BC biopsies collected before and after endocrine-therapy, revealed that endocrine-resistant tumors are enriched in amplification/mutation of FGFR family members (61). *In vitro*, activation of FGFR pathway, either by addition of FGF ligand in the culture medium or by FGFR overexpression, induced cross-resistance to Palbociclib and endocrine-therapy in HR+ BC cell lines. This effect was mediated by activation of Ras-MEK-ERK pathway and thus efficiently counteracted by administration of either MEK- or FGFR-inhibitors (61). Importantly, different FGFR inhibitors showed variable degrees of efficacy, depending on which member of FGFR was mutated and what type of mutation was found. This finding suggests the need for the development of different therapeutic strategies based on the FGFR specific status to overcome FGFR-induced drug resistance.

Recent data collected by our group also supports the interaction between CDK-i and EGFR pathway, in HR+ BC, *via* the modulation of miR-223. Treatment with Palbociclib restrains E2F1 transcriptional activity, which normally acts as a repressor of the miR-223 promoter, thereby restoring miR-223 expression. Since miR-223 then targets EGF expression, Palbociclib treatment eventually results in autocrine and paracrine dampening of EGFR pathway in tumor cells as well as in the tumor microenvironment (44, 92).

Not only the use of RTK-inhibitors may represent a valuable strategy to counteract acquired resistance to CDK-i, but also the concomitant administration of CDK-i and RTK-i is strongly encouraged for the treatment of naïve tumors. In esophageal squamous cell carcinoma, CDK-i attenuated cell growth in monotherapy, but was more effective in combination with pan-ERBB inhibitor, afatinib (62). In a PDX model of pediatric neuroblastoma harboring mutations of anaplastic lymphoma kinase (ALK), CDK-i synergized with the ALK inhibitor, Ceritinib, inducing cell-cycle arrest and cell death (63).

## CDK-i and Immune Checkpoint Inhibition

Several recent studies highlight that treatment with CDK-i may induce an immunomodulation of the tumor, thus suggesting the possibility to combine CDK-i with immune checkpoint inhibitors. Among the first evidences of a direct interaction between CDK-i and immunomodulation, there is the observation that CDK4 regulates PD-L1 protein stability and that combining CDK-i treatment with anti-PD-1 immunotherapy enhanced tumor regression and improved overall survival rates in mouse tumor models (64). Since then, many other studies supported an interaction between immunotherapy and CDK-I activity. As discussed above, mTOR activation plays a central role in determining CDK-i resistance. However, a recent study puts mTOR pathway in a different light, highlighting how it may indirectly reinforce the lymphocytic response against tumor cells, eventually contributing to CDK-i efficacy. Under CDK-i treatment, an increase in cellular metabolic activity is registered in neoplastic cells, mainly due to the dysregulation of PI3K/mTOR pathway (93). As a consequence, neoplastic cells become hypertrophic, increase their mitochondrial content and oxidative stress. These processes eventually determine the production of chemokines, like CCL5, CXCL9 and CXCL10, eventually leading to a greater T cell recruitment in the tumor microenvironment (93).

By indirectly reducing E2F activity, CDK-i can lead to a decrease of DNA methyl-transferase-1 (DNMT1) expression in neoplastic cells and, as a consequence, re-expression of endogenous retroviral genes by reduced methylation. This event closely mimics a viral process and results in activation of the interferon (IFN) signaling pathway, antigen presentation and recruitment of cytotoxic T cells in tumor microenvironment. However, DNMT1 also limits the expression of p21 by methylation of its coding gene CDKN1A in T-reg cells. Therefore, the inhibition of E2F-DNMT1 axis by CDK-i administration acts on one side on tumor cells, making them more antigenic, but, on the other, also on T-reg cells in tumor microenvironment, reducing their number by increasing p21 expression (94). These findings have been recently extended in a study that has demonstrated a positive modulation of antigen presentation mechanisms induced by Abemaciclib and a reciprocal synergy with anti-PD-L1 therapy (65).

Despite other small inhibitors, like MEK-inhibitors, have demonstrated only a transient benefit in combination with PD1-blockade, due to the unwanted prevention of priming of naïve T cells, Abemaciclib, probably thanks to its minor effect on CDK6 compared to CDK4, showed limited suppression of T-cells (65).

Moreover, it has been demonstrated that the immunogenic effect of CDK-i can be enhanced by concomitant administration of PI3K inhibitors. As previously mentioned, combination of PI3K-i and CDK-i has been demonstrated to be effective in preventing insurgence of resistance and inducing apoptosis (23). Moreover, in an *in vitro* model of TNBC, dual treatment with Alpelisib/Ribociclib evoked an increased tumor immunogenicity due to overexpression of both HLA antigens and CTLA-4 on tumor cell-surface and decreased expression of PD-L1, which is known to be regulated by the PI3K pathway (95). Therefore, double inhibition of PI3K and CDK4/6 in a syngeneic mouse model of TNBC was followed by a switch in the tumor-associated immune cells, with an increase in T-cells and mature NK cells, and a decrease of immunosuppressive monocytic myeloid-derived suppressor cells (mMDSC) and Tregs. As a consequence, combined inhibition of PI3K and CDK4/6, along with monoclonal antibody against immune checkpoints like PD-1 and CTLA-4, induced complete and durable regression of established TNBC mouse tumors, *in vivo* (60).

In BC, a transcriptomic analysis of parental cell lines and their endocrine-resistant and Palbociclib resistant derivatives, revealed that CDK-i resistant phenotype inversely correlated with an aberrant IFN/STAT1 pathway (96). Furthermore, a so called IFN-related Palbociclib-resistance signature was identified and validated in two neoadjuvant trials, in which patients were treated with CDK-i and endocrine therapy (96). Considering that IFNγ signaling pathway induces the expression of immune checkpoints, such as PD-L1 and CTLA-4, these results support once more the possibility to administer anti-immune checkpoint compound in combination with CDK-i, not only to improve the efficacy of CDK-i but also to tackle CDK-i resistance.

## CDK-i and Autophagy Inhibition

As mentioned before, CDK4/6 inhibition triggers both cell cycle arrest and senescence. As a stress tolerance mechanism, neoplastic cells may activate autophagy, a catabolic process of cellular recycle that reduces reactive oxygen species (ROS), produces energy for cell survival and mediates resistance to several therapeutics. BC cell lines treated with low doses of Palbociclib preserved an intact autophagic flux and relied on its activation for reverting G1 arrest. In this context, autophagy inhibitors, like chloroquine and hydroxychloroquine, synergized with CDK-i inducing ROS accumulation, growth inhibition, irreversible G1 arrest and then senescence. Interestingly, combination of CDK-i and autophagy-inhibitors was also effective in other tumor cell lines, if RB1 was intact and no oncogenic form of cyclin E was present (16). To note, as much as CDK-i, also MEKi elicited resistance mechanisms mediated by autophagy that were efficiently counteracted by administration of autophagy inhibitors (45).

## CDK-i and Hippo Pathway Inhibition

The Hippo pathway is dysregulated in different cancer types (46) and the alteration of YAP, a Hippo pathway effector, leads to overexpression and activation of CDK6. Therefore, it is not surprising that YAP inhibitors have been proposed in combination therapy with CDK-i, although very little is known about the effects of this combination, so far. One study reported that combination of CDK-i with YAP inhibitors counteracted tumor growth and overcame radiation-resistance in esophageal cancer models (66). Another study reported that chronic treatment with Abemaciclib did not decrease the sensitivity to YAP inhibitors in pancreatic cancer cells (67).

It has also been reported that the activation of the Hippo pathway may confer resistance to CDK-i. In fact, a genomic analysis performed on CDK-i resistant HR+ BC samples identified loss of FAT1, member of the cadherin superfamily that exerts a regulatory role on Hippo pathway, as a common event. FAT1 loss caused YAP activation, CDK6 upregulation and resistance to CDK-i (97). Altogether, these results strongly support the need for further testing the combination of CDK-i and YAP-inhibitors.

## CDK-i and AP-1 Inhibition

Activator protein-1 (AP-1) is a heterodimeric transcription factor, primarily composed of proteins belonging to the Fos and Jun families that exerts a regulatory role on many physiological and pathological processes (98). In the past 10 years, small molecules able to inhibit the chromatin-binding capability of Fos proteins have been developed, showing interesting results in the therapy of both inflammatory disease and cancer (99). In BC cells, administration of Palbociclib reduces expression and activation of c-Jun, followed by a reduction of EMT-associated proteins expression (98). Therefore, Palbociclib administration impinges on motility, invasive capability and metastatic potential of BC cells. However, another study reports that CDK-i resistant cells show increasing levels of c-Fos and c-Jun that, in turn, determine a higher AP-1 transcriptional activity responsible for, at least in part, the resistant phenotype insurgence (100). In this model, AP-1 blockade strongly inhibits the growth of CDK-i resistant cells (100).

The possible interplay between CDK-i and AP-1 activity appears even more interesting when AP-1 functions are taken into account. On one hand, AP-1 is responsible for the activation of therapy-induced senescence program in neoplastic cells that, as we discussed above, represents one of the main consequences of Palbociclib administration (101). On the other, the aberrant expression of AP-1 also determines a re-wiring of the transcriptional program of BC cells, promoting cellular plasticity and resistance to endocrine-therapy, an event closely connected to the CDK-i resistant phenotype (102). Altogether, these data support the central role of AP-1 in the adaptive strategy of neoplastic cells under CDK-i treatment and provide a rationale for the combined administration of CDK- and AP-1 inhibitors.

## Conclusions

The introduction of CDK-i has drastically changed the clinical approach to HR+ BC and their use is expected to be extended to many other tumor types. If we consider that CDK-i induce growth arrest but not apoptosis and that intrinsic and acquired resistance are common events in clinical practice, combining CDK-i with other compounds appears to be a reasonable and necessary strategy, in order to improve their efficacy and prevent or revert a resistant phenotype.

In this context, it is now clear that, when administered in combination with conventional chemotherapy, their efficacy strictly depends on the treatment schedule, since only CDK-i administration after the chemotherapeutic drug may refrain neoplastic cells recovery and results in a synergistic action. Otherwise, cell-cycle blockade induced by CDK-i may even protect tumor cells from the cytotoxic effects of the chemotherapy.

Combination of CDK-i with other targeted therapies is still an emerging but rapidly expanding field. At least in preclinical models, several inhibitors have shown to improve CDK-i sensitivity, counteracting the mechanisms responsible for the CDK-i resistant phenotype. In this context it will be of outmost importance to precisely identify the patients who might benefit from specific combination therapies, identifying reliable biomarkers. Similarly, promising evidences support the possibility that CDK-i might potentiate the activity of immunotherapy with checkpoint inhibitors. Again, which patients might benefit from these new combinations has still to be precisely defined.

We have to face the emerging notion that the phenotype of CDK-i resistance is not a permanent status, but a dynamic process in which at least two steps, not necessarily consequent one to another, are definable. Therefore, targeting CDK-i resistance still appears a challenging task to be achieved. Rb inactivation is often involved in this process and, to date, it remains a major limit for CDK-i administration, further raising the question of how it will be possible to bypass this type of genomic alteration and restore Rb functionality.

Further, if the status of CDK-i resistance can not be successfully targeted, more effort should be made to prevent the onset of CDK-i resistance. This will be possible by the precocious identification of the mechanisms on which the resistance relies, that will lead to the prompt identification of tumors that will become potentially resistant. The identification of such escaping pathways could lead the choice of the best companion for CDK-i, to achieve a more successful combined therapy.

## Author Contributions

Conceptualization, GLRV, GB, and BB. Writing-original draft preparation, GLRV, and BB. Writing-review and editing, GLRV, MS, IS, ADA, AV, GB, and BB. All authors contributed to the article and approved the submitted version.

## Funding

This work was supported by Fondazione AIRC per la Ricerca sul Cancro (AIRC), IG#20061 to BB, MFAG# 24321 to MS and IG#26253 to GB, by Ministero della Salute, PE-2016-02361040 to GB, by Ministero dell’Università e della Ricerca, PON ARS01_00568 to GB.

## Conflict of Interest

The authors declare that the research was conducted in the absence of any commercial or financial relationships that could be construed as a potential conflict of interest.

## Publisher’s Note

All claims expressed in this article are solely those of the authors and do not necessarily represent those of their affiliated organizations, or those of the publisher, the editors and the reviewers. Any product that may be evaluated in this article, or claim that may be made by its manufacturer, is not guaranteed or endorsed by the publisher.

## References

[1] Nasmyth K. A Prize for Proliferation. Cell (2001) 107:689–701. doi: 10.1016/S0092-8674(01)00604-3 11747804

[2] Asghar U Witkiewicz AK Turner NC Knudsen ES. The History and Future of Targeting Cyclin-Dependent Kinases in Cancer Therapy. Nat Rev Drug Discov (2015) 14:130–46. doi: 10.1038/nrd4504 PMC4480421 25633797

[3] Choi YJ Li X Hydbring P Sanda T Stefano J Christie AL. The Requirement for Cyclin D Function in Tumor Maintenance. Cancer Cell (2012) 22:438–51. doi: 10.1016/j.ccr.2012.09.015 PMC3487466 23079655

[4] Toogood PL. Cyclin-Dependent Kinase Inhibitors for Treating Cancer. Med Res Rev (2001) 21:487–98. doi: 10.1002/med.1021 11607930

[5] Toogood PL Harvey PJ Repine JT Sheehan DJ VanderWel SN Zhou H. Discovery of a Potent and Selective Inhibitor of Cyclin-Dependent Kinase 4/6. J Med Chem (2005) 48:2388–406. doi: 10.1021/jm049354h 15801831

[6] Baughn LB Di Liberto M Wu K Toogood PL Louie T Gottschalk R. A Novel Orally Active Small Molecule Potently Induces G1 Arrest in Primary Myeloma Cells and Prevents Tumor Growth by Specific Inhibition of Cyclin-Dependent Kinase 4/6. Cancer Res (2006) 66:7661–7. doi: 10.1158/0008-5472.CAN-06-1098 16885367

[7] Finn RS Martin M Rugo HS Jones S Im S-A Gelmon K. Palbociclib and Letrozole in Advanced Breast Cancer. N Engl J Med (2016) 375:1925–36. doi: 10.1056/NEJMoa1607303 27959613

[8] Hortobagyi GN Stemmer SM Burris HA Yap Y-S Sonke GS Paluch-Shimon S. Ribociclib as First-Line Therapy for HR-Positive, Advanced Breast Cancer. N Engl J Med (2016) 375:1738–48. doi: 10.1056/NEJMoa1609709 27717303

[9] Goetz MP Toi M Campone M Sohn J Paluch-Shimon S Huober J. MONARCH 3: Abemaciclib As Initial Therapy for Advanced Breast Cancer. J Clin Oncol Off J Am Soc Clin Oncol (2017) 35:3638–46. doi: 10.1200/JCO.2017.75.6155 28968163

[10] Álvarez-Fernández M Malumbres M. Mechanisms of Sensitivity and Resistance to CDK4/6 Inhibition. Cancer Cell (2020) 37:514–29. doi: 10.1016/j.ccell.2020.03.010 32289274

[11] Fassl A Geng Y Sicinski P. CDK4 and CDK6 Kinases: From Basic Science to Cancer Therapy. Science (2022) 375, eabc1495. doi: 10.1126/science.abc1495 35025636 PMC9048628

[12] Liu F Korc M. Cdk4/6 Inhibition Induces Epithelial–Mesenchymal Transition and Enhances Invasiveness in Pancreatic Cancer Cells. Mol Cancer Ther (2012) 11:2138–48. doi: 10.1158/1535-7163.MCT-12-0562 PMC3752412 22869556

[13] Kettner NM Vijayaraghavan S Durak MG Bui T Kohansal M Ha MJ. Combined Inhibition of STAT3 and DNA Repair in Palbociclib-Resistant ER-Positive Breast Cancer. Clin Cancer Res Off J Am Assoc Cancer Res (2019) 25:3996–4013. doi: 10.1158/1078-0432.CCR-18-3274 PMC6606366 30867218

[14] Klein ME Kovatcheva M Davis LE Tap WD Koff A. CDK4/6 Inhibitors: The Mechanism of Action May Not Be as Simple as Once Thought. Cancer Cell (2018) 34:9–20. doi: 10.1016/j.ccell.2018.03.023 29731395 PMC6039233

[15] Ogata R Kishino E Saitoh W Koike Y Kurebayashi J. Resistance to Cyclin-Dependent Kinase (CDK) 4/6 Inhibitors Confers Cross-Resistance to Other CDK Inhibitors But Not to Chemotherapeutic Agents in Breast Cancer Cells. Breast Cancer Tokyo Jpn (2021) 28:206–15. doi: 10.1007/s12282-020-01150-8 PMC7796879 32860163

[16] Vijayaraghavan S Karakas C Doostan I Chen X Bui T Yi M. CDK4/6 and Autophagy Inhibitors Synergistically Induce Senescence in Rb Positive Cytoplasmic Cyclin E Negative Cancers. Nat Commun (2017) 8:15916. doi: 10.1038/ncomms15916 28653662 PMC5490269

[17] Sumi NJ Kuenzi BM Knezevic CE Remsing Rix LL Rix U. Chemoproteomics Reveals Novel Protein and Lipid Kinase Targets of Clinical CDK4/6 Inhibitors in Lung Cancer. ACS Chem Biol (2015) 10:2680–6. doi: 10.1021/acschembio.5b00368 PMC4684772 26390342

[18] Miettinen TP Peltier J Härtlova A Gierliński M Jansen VM Trost M. Thermal Proteome Profiling of Breast Cancer Cells Reveals Proteasomal Activation by CDK4/6 Inhibitor Palbociclib. EMBO J (2018) 37:e98359. doi: 10.15252/embj.201798359 29669860 PMC5978322

[19] Rubio C Martínez-Fernández M Segovia C Lodewijk I Suarez-Cabrera C Segrelles C. CDK4/6 Inhibitor as a Novel Therapeutic Approach for Advanced Bladder Cancer Independently of *RB1* Status. Clin Cancer Res (2019) 25:390–402. doi: 10.1158/1078-0432.CCR-18-0685 30242024

[20] Portman N Alexandrou S Carson E Wang S Lim E Caldon CE. Overcoming CDK4/6 Inhibitor Resistance in ER-Positive Breast Cancer. Endocr Relat Cancer (2019) 26:R15–30. doi: 10.1530/ERC-18-0317 30389903

[21] Iida M Toyosawa D Nakamura M Tsuboi K Tokuda E Niwa T. Decreased ER Dependency After Acquired Resistance to CDK4/6 Inhibitors. Breast Cancer (2020) 27:963–72. doi: 10.1007/s12282-020-01090-3 32297248

[22] Michaloglou C Crafter C Siersbaek R Delpuech O Curwen JO Carnevalli LS. Combined Inhibition of mTOR and CDK4/6 Is Required for Optimal Blockade of E2F Function and Long-Term Growth Inhibition in Estrogen Receptor–positive Breast Cancer. Mol Cancer Ther (2018) 17:908–20. doi: 10.1158/1535-7163.MCT-17-0537 PMC6485624 29483206

[23] Herrera-Abreu MT Palafox M Asghar U Rivas MA Cutts RJ Garcia-Murillas I. Early Adaptation and Acquired Resistance to CDK4/6 Inhibition in Estrogen Receptor-Positive Breast Cancer. Cancer Res (2016) 76:2301–13. doi: 10.1158/0008-5472.CAN-15-0728 PMC5426059 27020857

[24] Olmez I Brenneman B Xiao A Serbulea V Benamar M Zhang Y. Combined CDK4/6 and mTOR Inhibition Is Synergistic Against Glioblastoma *Via* Multiple Mechanisms. Clin Cancer Res Off J Am Assoc Cancer Res (2017) 23:6958–68. doi: 10.1158/1078-0432.CCR-17-0803 PMC5690820 28814434

[25] Olanich ME Sun W Hewitt SM Abdullaev Z Pack SD Barr FG. CDK4 Amplification Reduces Sensitivity to CDK4/6 Inhibition in Fusion-Positive Rhabdomyosarcoma. Clin Cancer Res Off J Am Assoc Cancer Res (2015) 21:4947–59. doi: 10.1158/1078-0432.CCR-14-2955 PMC4583342 25810375

[26] Otto T Sicinski P. Cell Cycle Proteins as Promising Targets in Cancer Therapy. Nat Rev Cancer (2017) 17:93–115. doi: 10.1038/nrc.2016.138 28127048 PMC5345933

[27] Yang C Li Z Bhatt T Dickler M Giri D Scaltriti M. Acquired CDK6 Amplification Promotes Breast Cancer Resistance to CDK4/6 Inhibitors and Loss of ER Signaling and Dependence. Oncogene (2017) 36:2255–64. doi: 10.1038/onc.2016.379 PMC5393973 27748766

[28] Landis MW Pawlyk BS Li T Sicinski P Hinds PW. Cyclin D1-Dependent Kinase Activity in Murine Development and Mammary Tumorigenesis. Cancer Cell (2006) 9:13–22. doi: 10.1016/j.ccr.2005.12.019 16413468

[29] Perou CM Sørlie T Eisen MB van de Rijn M Jeffrey SS Rees CA. Molecular Portraits of Human Breast Tumours. Nature (2000) 406:747–52. doi: 10.1038/35021093 10963602

[30] Harbeck N Penault-Llorca F Cortes J Gnant M Houssami N Poortmans P. Breast Cancer. Nat Rev Dis Primer (2019) 5:66. doi: 10.1038/s41572-019-0111-2 31548545

[31] Finn RS Dering J Conklin D Kalous O Cohen DJ Desai AJ. PD 0332991, a Selective Cyclin D Kinase 4/6 Inhibitor, Preferentially Inhibits Proliferation of Luminal Estrogen Receptor-Positive Human Breast Cancer Cell Lines In Vitro . Breast Cancer Res BCR (2009) 11:R77. doi: 10.1186/bcr2419 PMC2790859 19874578

[32] Roberts PJ Bisi JE Strum JC Combest AJ Darr DB Usary JE. Multiple Roles of Cyclin-Dependent Kinase 4/6 Inhibitors in Cancer Therapy. J Natl Cancer Inst (2012) 104:476–87. doi: 10.1093/jnci/djs002 PMC3309128 22302033

[33] Wang L Wang J Blaser BW Duchemin A-M Kusewitt DF Liu T. Pharmacologic Inhibition of CDK4/6: Mechanistic Evidence for Selective Activity or Acquired Resistance in Acute Myeloid Leukemia. Blood (2007) 110:2075–83. doi: 10.1182/blood-2007-02-071266 17537993

[34] Johnson SM Torrice CD Bell JF Monahan KB Jiang Q Wang Y. Mitigation of Hematologic Radiation Toxicity in Mice Through Pharmacological Quiescence Induced by CDK4/6 Inhibition. J Clin Invest (2010) 120:2528–36. doi: 10.1172/JCI41402 PMC2898594 20577054

[35] Chu I Sun J Arnaout A Kahn H Hanna W Narod S. P27 Phosphorylation by Src Regulates Inhibition of Cyclin E-Cdk2. Cell (2007) 128:281–94. doi: 10.1016/j.cell.2006.11.049 PMC1961623 17254967

[36] Sledge GW Jr Toi M Neven P Sohn J Inoue K Pivot X. The Effect of Abemaciclib Plus Fulvestrant on Overall Survival in Hormone Receptor–Positive, ERBB2-Negative Breast Cancer That Progressed on Endocrine Therapy—MONARCH 2: A Randomized Clinical Trial. JAMA Oncol (2020) 6:116–24. doi: 10.1001/jamaoncol.2019.4782 PMC6777264 31563959

[37] Rampioni Vinciguerra GL Citron F Segatto I Belletti B Vecchione A Baldassarre G. P27kip1 at the Crossroad Between Actin and Microtubule Dynamics. Cell Div (2019) 14:2. doi: 10.1186/s13008-019-0045-9 30976290 PMC6442415

[38] Guiley KZ Stevenson JW Lou K Barkovich KJ Kumarasamy V Wijeratne TU. P27 Allosterically Activates Cyclin-Dependent Kinase 4 and Antagonizes Palbociclib Inhibition. Science (2019) 366:eaaw2106. doi: 10.1126/science.aaw2106 31831640 PMC7592119

[39] Grimmler M Wang Y Mund T Cilensek Z Keidel E-M Waddell MB. Cdk-Inhibitory Activity and Stability of p27Kip1 Are Directly Regulated by Oncogenic Tyrosine Kinases. Cell (2007) 128:269–80. doi: 10.1016/j.cell.2006.11.047 17254966

[40] O’Leary B Cutts RJ Liu Y Hrebien S Huang X Fenwick K. The Genetic Landscape and Clonal Evolution of Breast Cancer Resistance to Palbociclib Plus Fulvestrant in the PALOMA-3 Trial. Cancer Discov (2018) 8:1390–403. doi: 10.1158/2159-8290.CD-18-0264 PMC6368247 30206110

[41] Hayes TK Luo F Cohen O Goodale AB Lee Y Pantel S. A Functional Landscape of Resistance to MEK1/2 and CDK4/6 Inhibition in NRAS-Mutant Melanoma. Cancer Res (2019) 79:2352–66. doi: 10.1158/0008-5472.CAN-18-2711 PMC7227487 30819666

[42] Haines E Chen T Kommajosyula N Chen Z Herter-Sprie GS Cornell L. Palbociclib Resistance Confers Dependence on an FGFR-MAP Kinase-mTOR-Driven Pathway in KRAS-Mutant Non-Small Cell Lung Cancer. Oncotarget (2018) 9:31572–89. doi: 10.18632/oncotarget.25803 PMC6114982 30167080

[43] Sobhani N Fassl A Mondani G Generali D Otto T. Targeting Aberrant FGFR Signaling to Overcome CDK4/6 Inhibitor Resistance in Breast Cancer. Cells (2021) 10:293. doi: 10.3390/cells10020293 33535617 PMC7912842

[44] Fabris L Berton S Citron F D’Andrea S Segatto I Nicoloso MS. Radiotherapy-Induced miR-223 Prevents Relapse of Breast Cancer by Targeting the EGF Pathway. Oncogene (2016) 35:4914–26. doi: 10.1038/onc.2016.23 PMC5028421 26876200

[45] Kinsey CG Camolotto SA Boespflug AM Guillen KP Foth M Truong A. Protective Autophagy Elicited by RAF→MEK→ERK Inhibition Suggests a Treatment Strategy for RAS-Driven Cancers. Nat Med (2019) 25:620–7. doi: 10.1038/s41591-019-0367-9 PMC6452642 30833748

[46] Li Z Zou W Zhang J Zhang Y Xu Q Li S. Mechanisms of CDK4/6 Inhibitor Resistance in Luminal Breast Cancer. Front Pharmacol (2020) 11:580251. doi: 10.3389/fphar.2020.580251 33364954 PMC7751736

[47] Turner NC Slamon DJ Ro J Bondarenko I Im S-A Masuda N. Overall Survival With Palbociclib and Fulvestrant in Advanced Breast Cancer. N Engl J Med (2018) 379:1926–36. doi: 10.1056/NEJMoa1810527 30345905

[48] Slamon DJ Neven P Chia S Fasching PA De Laurentiis M Im S-A. Overall Survival With Ribociclib Plus Fulvestrant in Advanced Breast Cancer. N Engl J Med (2020) 382:514–24. doi: 10.1056/NEJMoa1911149 31826360

[49] Im S-A Lu Y-S Bardia A Harbeck N Colleoni M Franke F. Overall Survival With Ribociclib Plus Endocrine Therapy in Breast Cancer. N Engl J Med (2019) 381:307–16. doi: 10.1056/NEJMoa1903765 31166679

[50] Dall’Acqua A Sonego M Pellizzari I Pellarin I Canzonieri V D’Andrea S. CDK6 Protects Epithelial Ovarian Cancer From Platinum-Induced Death *Via* FOXO3 Regulation. EMBO Mol Med (2017) 9:1415–33. doi: 10.15252/emmm.201607012 PMC5623833 28778953

[51] Iyengar M O’Hayer P Cole A Sebastian T Yang K Coffman L. CDK4/6 Inhibition as Maintenance and Combination Therapy for High Grade Serous Ovarian Cancer. Oncotarget (2018) 9:15658–72. doi: 10.18632/oncotarget.24585 PMC5884655 29644000

[52] Salvador-Barbero B Álvarez-Fernández M Zapatero-Solana E El Bakkali A Menéndez MDC López-Casas PP. CDK4/6 Inhibitors Impair Recovery From Cytotoxic Chemotherapy in Pancreatic Adenocarcinoma. Cancer Cell (2020) 37:340–53.e6. doi: 10.1016/j.ccell.2020.01.007 32109375

[53] Francis AM Alexander A Liu Y Vijayaraghavan S Low KH Yang D. CDK4/6 Inhibitors Sensitize Rb-Positive Sarcoma Cells to Wee1 Kinase Inhibition Through Reversible Cell Cycle Arrest. Mol Cancer Ther (2017) 16:1751–64. doi: 10.1158/1535-7163.MCT-17-0040 PMC5975955 28619757

[54] Fallah Y Demas DM Jin L He W Shajahan-Haq AN. Targeting WEE1 Inhibits Growth of Breast Cancer Cells That Are Resistant to Endocrine Therapy and CDK4/6 Inhibitors. Front Oncol (2021) 11:681530. doi: 10.3389/fonc.2021.681530 34277427 PMC8281892

[55] AbuHammad S Cullinane C Martin C Bacolas Z Ward T Chen H. Regulation of PRMT5–MDM4 Axis is Critical in the Response to CDK4/6 Inhibitors in Melanoma. Proc Natl Acad Sci (2019) 116:17990–8000. doi: 10.1073/pnas.1901323116 PMC6731642 31439820

[56] Vilgelm AE Saleh N Shattuck-Brandt R Riemenschneider K Slesur L Chen S-C. MDM2 Antagonists Overcome Intrinsic Resistance to CDK4/6 Inhibition by Inducing P21. Sci Transl Med (2019) 11:eaav7171. doi: 10.1126/scitranslmed.aav7171 31413145 PMC7584132

[57] Rampioni Vinciguerra GL Dall’Acqua A Segatto I Mattevi MC Russo F Favero A. P27kip1 Expression and Phosphorylation Dictate Palbociclib Sensitivity in KRAS-Mutated Colorectal Cancer. Cell Death Dis (2021) 12:951. doi: 10.1038/s41419-021-04241-2 34654798 PMC8519959

[58] Luo C-W Hou M-F Huang C-W Wu C-C Ou-Yang F Li Q-L. The CDK6-C-Jun-Sp1-MMP-2 Axis as a Biomarker and Therapeutic Target for Triple-Negative Breast Cancer. Am J Cancer Res (2020) 10:4325–41. PMC7783751 33415002

[59] de Leeuw R McNair CM Schiewer MJ Neupane NP Brand LJ Augello MA. MAPK Reliance *Via* Acquired CDK4/6 Inhibitor Resistance in Cancer. Clin Cancer Res Off J Am Assoc Cancer Res (2018) 24:4201–14. doi: 10.1158/1078-0432.CCR-18-0410 PMC6125187 29739788

[60] Teo ZL Versaci S Dushyanthen S Caramia F Savas P Mintoff CP. Combined CDK4/6 and PI3Kα Inhibition Is Synergistic and Immunogenic in Triple-Negative Breast Cancer. Cancer Res (2017) 77:6340–52. doi: 10.1158/0008-5472.CAN-17-2210 28947417

[61] Mao P Cohen O Kowalski KJ Kusiel JG Buendia-Buendia JE Cuoco MS. Acquired FGFR and FGF Alterations Confer Resistance to Estrogen Receptor (ER) Targeted Therapy in ER+ Metastatic Breast Cancer. Clin Cancer Res (2020) 26:5974–89. doi: 10.1158/1078-0432.CCR-19-3958 32723837

[62] Zhou J Wu Z Zhang Z Goss L McFarland J Nagaraja A. Pan-ERBB Kinase Inhibition Augments CDK4/6 Inhibitor Efficacy in Oesophageal Squamous Cell Carcinoma. Gut (2021) gutjnl-2020-323276. doi: 10.1136/gutjnl-2020-323276 PMC8921580 33789967

[63] Wood AC Krytska K Ryles HT Infarinato NR Sano R Hansel TD. Dual ALK and CDK4/6 Inhibition Demonstrates Synergy Against Neuroblastoma. Clin Cancer Res Off J Am Assoc Cancer Res (2017) 23:2856–68. doi: 10.1158/1078-0432.CCR-16-1114 PMC5457336 27986745

[64] Zhang J Bu X Wang H Zhu Y Geng Y Nihira NT. Cyclin D-CDK4 Kinase Destabilizes PD-L1 *Via* Cullin 3-SPOP to Control Cancer Immune Surveillance. Nature (2018) 553:91–5. doi: 10.1038/nature25015 PMC5754234 29160310

[65] Schaer DA Beckmann RP Dempsey JA Huber L Forest A Amaladas N. The CDK4/6 Inhibitor Abemaciclib Induces a T Cell Inflamed Tumor Microenvironment and Enhances the Efficacy of PD-L1 Checkpoint Blockade. Cell Rep (2018) 22:2978–94. doi: 10.1016/j.celrep.2018.02.053 29539425

[66] Li F Xu Y Liu B Singh PK Zhao W Jin J. YAP1-Mediated CDK6 Activation Confers Radiation Resistance in Esophageal Cancer - Rationale for the Combination of YAP1 and CDK4/6 Inhibitors in Esophageal Cancer. Clin Cancer Res Off J Am Assoc Cancer Res (2019) 25:2264–77. doi: 10.1158/1078-0432.CCR-18-1029 30563933

[67] Dhir T Schultz CW Jain A Brown SZ Haber A Goetz A. Abemaciclib Is Effective Against Pancreatic Cancer Cells and Synergizes With HuR and YAP1 Inhibition. Mol Cancer Res (2019) 17:2029–41. doi: 10.1158/1541-7786.MCR-19-0589 PMC6794000 31383722

[68] Pandey K An H-J Kim SK Lee SA Kim S Lim SM. Molecular Mechanisms of Resistance to CDK4/6 Inhibitors in Breast Cancer: A Review. Int J Cancer (2019) 145:1179–88. doi: 10.1002/ijc.32020 PMC6767051 30478914

[69] Roberts PJ Kumarasamy V Witkiewicz AK Knudsen ES. Chemotherapy and CDK4/6 Inhibitors: Unexpected Bedfellows. Mol Cancer Ther (2020) 19:1575–88. doi: 10.1158/1535-7163.MCT-18-1161 PMC7473501 32546660

[70] Wiedemeyer WR Dunn IF Quayle SN Zhang J Chheda MG Dunn GP. Pattern of Retinoblastoma Pathway Inactivation Dictates Response to CDK4/6 Inhibition in GBM. Proc Natl Acad Sci (2010) 107:11501–6. doi: 10.1073/pnas.1001613107 PMC2895056 20534551

[71] Yu Q Geng Y Sicinski P. Specific Protection Against Breast Cancers by Cyclin D1 Ablation. Nature (2001) 411:1017–21. doi: 10.1038/35082500 11429595

[72] Yu Q Sicinska E Geng Y Ahnström M Zagozdzon A Kong Y. Requirement for CDK4 Kinase Function in Breast Cancer. Cancer Cell (2006) 9:23–32. doi: 10.1016/j.ccr.2005.12.012 16413469

[73] Prat A Perou CM. Deconstructing the Molecular Portraits of Breast Cancer. Mol Oncol (2011) 5:5–23. doi: 10.1016/j.molonc.2010.11.003 21147047 PMC5528267

[74] Fry DW Harvey PJ Keller PR Elliott WL Meade M Trachet E. Specific Inhibition of Cyclin-Dependent Kinase 4/6 by PD 0332991 and Associated Antitumor Activity in Human Tumor Xenografts. Mol Cancer Ther (2004) 3:1427–38. 15542782

[75] Pernas S Tolaney SM Winer EP Goel S. CDK4/6 Inhibition in Breast Cancer: Current Practice and Future Directions. Ther Adv Med Oncol (2018) 10:1758835918786451. doi: 10.1177/1758835918786451 30038670 PMC6050811

[76] Fribbens C O’Leary B Kilburn L Hrebien S Garcia-Murillas I Beaney M. Plasma ESR1 Mutations and the Treatment of Estrogen Receptor–Positive Advanced Breast Cancer. J Clin Oncol (2016) 34:2961–8. doi: 10.1200/JCO.2016.67.3061 27269946

[77] Haricharan S Punturi N Singh P Holloway KR Anurag M Schmelz J. Loss of MutL Disrupts CHK2-Dependent Cell-Cycle Control Through CDK4/6 to Promote Intrinsic Endocrine Therapy Resistance in Primary Breast Cancer. Cancer Discovery (2017) 7:1168–83. doi: 10.1158/2159-8290.CD-16-1179 PMC5733075 28801307

[78] Caldon CE Sergio CM Kang J Muthukaruppan A Boersma MN Stone A. Cyclin E2 Overexpression Is Associated With Endocrine Resistance But Not Insensitivity to CDK2 Inhibition in Human Breast Cancer Cells. Mol Cancer Ther (2012) 11:1488–99. doi: 10.1158/1535-7163.MCT-11-0963 22564725

[79] Turner NC Liu Y Zhu Z Loi S Colleoni M Loibl S. Cyclin E1 Expression and Palbociclib Efficacy in Previously Treated Hormone Receptor-Positive Metastatic Breast Cancer. J Clin Oncol Off J Am Soc Clin Oncol (2019) 37:1169–78. doi: 10.1200/JCO.18.00925 PMC6506420 30807234

[80] Formisano L Lu Y Servetto A Hanker AB Jansen VM Bauer JA. Aberrant FGFR Signaling Mediates Resistance to CDK4/6 Inhibitors in ER+ Breast Cancer. Nat Commun (2019) 10:1373. doi: 10.1038/s41467-019-09068-2 30914635 PMC6435685

[81] Dall’Acqua A Bartoletti M Masoudi-Khoram N Sorio R Puglisi F Belletti B. Inhibition of CDK4/6 as Therapeutic Approach for Ovarian Cancer Patients: Current Evidences and Future Perspectives. Cancers (2021) 13:3035. doi: 10.3390/cancers13123035 34204543 PMC8235237

[82] Franco J Witkiewicz AK Knudsen ES. CDK4/6 Inhibitors Have Potent Activity in Combination With Pathway Selective Therapeutic Agents in Models of Pancreatic Cancer. Oncotarget (2014) 5:6512–25. doi: 10.18632/oncotarget.2270 PMC4171647 25156567

[83] McClendon AK Dean JL Rivadeneira DB Yu JE Reed CA Gao E. CDK4/6 Inhibition Antagonizes the Cytotoxic Response to Anthracycline Therapy. Cell Cycle Georget Tex (2012) 11:2747–55. doi: 10.4161/cc.21127 PMC3409014 22751436

[84] He S Roberts PJ Sorrentino JA Bisi JE Storrie-White H Tiessen RG. Transient CDK4/6 Inhibition Protects Hematopoietic Stem Cells From Chemotherapy-Induced Exhaustion. Sci Transl Med (2017) 9:eaal3986. doi: 10.1126/scitranslmed.aal3986 28446688 PMC5774632

[85] Vilgelm AE Pawlikowski JS Liu Y Hawkins OE Davis TA Smith J. Mdm2 and Aurora Kinase a Inhibitors Synergize to Block Melanoma Growth by Driving Apoptosis and Immune Clearance of Tumor Cells. Cancer Res (2015) 75:181–93. doi: 10.1158/0008-5472.CAN-14-2405 PMC4286469 25398437

[86] Gottesman SRS Somma J Tsiperson V Dresner L Govindarajulu U Patel P. Tyrosine Phosphorylation of p27Kip1 Correlates With Palbociclib Responsiveness in Breast Cancer Tumor Cells Grown in Explant Culture. Mol Cancer Res MCR (2019) 17:669–75. doi: 10.1158/1541-7786.MCR-18-0188 PMC6397661 30559257

[87] Patel P Asbach B Shteyn E Gomez C Coltoff A Bhuyan S. Brk/Protein Tyrosine Kinase 6 Phosphorylates P27kip1, Regulating the Activity of Cyclin D–Cyclin-Dependent Kinase 4. Mol Cell Biol (2015) 35:1506–22. doi: 10.1128/MCB.01206-14 PMC4387217 25733683

[88] Patel P Tsiperson V Gottesman SRS Somma J Blain SW. Dual Inhibition of CDK4 and CDK2 *Via* Targeting P27 Tyrosine Phosphorylation Induces a Potent and Durable Response in Breast Cancer Cells. Mol Cancer Res MCR (2018) 16:361–77. doi: 10.1158/1541-7786.MCR-17-0602 PMC5835198 29330290

[89] Wander SA Cohen O Gong X Johnson GN Buendia-Buendia JE Lloyd MR. The Genomic Landscape of Intrinsic and Acquired Resistance to Cyclin-Dependent Kinase 4/6 Inhibitors in Patients With Hormone Receptor–Positive Metastatic Breast Cancer. Cancer Discov (2020) 10:1174–93. doi: 10.1158/2159-8290.CD-19-1390 PMC8815415 32404308

[90] Lee MS Helms TL Feng N Gay J Chang QE Tian F. Efficacy of the Combination of MEK and CDK4/6 Inhibitors *In Vitro* and *In Vivo* in KRAS Mutant Colorectal Cancer Models. Oncotarget (2016) 7:39595–608. doi: 10.18632/oncotarget.9153 PMC5129956 27167191

[91] Formisano L Stauffer KM Young CD Bhola NE Guerrero AL Jansen VM. Association of FGFR1 With Erα Maintains Ligand-Independent ER Transcription and Mediates Resistance to Estrogen Deprivation in ER+ Breast Cancer. Clin Cancer Res Off J Am Assoc Cancer Res (2017) 23:6138–50. doi: 10.1158/1078-0432.CCR-17-1232 PMC6681458 28751448

[92] Citron F Segatto I Vinciguerra GLR Musco L Russo F Mungo G. Downregulation of miR-223 Expression Is an Early Event During Mammary Transformation and Confers Resistance to CDK4/6 Inhibitors in Luminal Breast Cancer. Cancer Res (2020) 80:1064–77. doi: 10.1158/0008-5472.CAN-19-1793 31862778

[93] Uzhachenko RV Bharti V Ouyang Z Blevins A Mont S Saleh N. Metabolic Modulation by CDK4/6 Inhibitor Promotes Chemokine-Mediated Recruitment of T Cells Into Mammary Tumors. Cell Rep (2021) 35:108944. doi: 10.1016/j.celrep.2021.108944 33826903 PMC8383195

[94] Goel S DeCristo MJ Watt AC BrinJones H Sceneay J Li BB. CDK4/6 Inhibition Triggers Anti-Tumor Immunity. Nature (2017) 548:471–5. doi: 10.1038/nature23465 PMC5570667 28813415

[95] Atefi M Avramis E Lassen A Wong D Robert L Foulad D. Effects of MAPK and PI3K Pathways on PD-L1 Expression in Melanoma. Clin Cancer Res Off J Am Assoc Cancer Res (2014) 20:3446–57. doi: 10.1158/1078-0432.CCR-13-2797 PMC4079734 24812408

[96] De Angelis C Fu X Cataldo ML Nardone A Pereira R Veeraraghavan J. Activation of the IFN Signaling Pathway Is Associated With Resistance to CDK4/6 Inhibitors and Immune Checkpoint Activation in ER-Positive Breast Cancer. Clin Cancer Res (2021) 27:4870–82. doi: 10.1158/1078-0432.CCR-19-4191 PMC8628647 33536276

[97] Li Z Razavi P Li Q Toy W Liu B Ping C. Loss of the FAT1 Tumor Suppressor Promotes Resistance to CDK4/6 Inhibitors *Via* the Hippo Pathway. Cancer Cell (2018) 34:893–905.e8. doi: 10.1016/j.ccell.2018.11.006 PMC6294301 30537512

[98] Qin G Xu F Qin T Zheng Q Shi D Xia W. Palbociclib Inhibits Epithelial-Mesenchymal Transition and Metastasis in Breast Cancer *Via* C-Jun/COX-2 Signaling Pathway. Oncotarget (2015) 6:41794–808. 10.18632/oncotarget.5993 PMC4747189 26540629

[99] Ye N Ding Y Wild C Shen Q Zhou J. Small Molecule Inhibitors Targeting Activator Protein 1 (AP-1). J Med Chem (2014) 57:6930–48. doi: 10.1021/jm5004733 PMC4148154 24831826

[100] Angelis CD Nardone A Cataldo ML Veeraraghavan J Fu X Giuliano M. Abstract P4-03-05: AP-1 as a Potential Mediator of Resistance to the Cyclin-Dependent Kinase (CDK) 4/6-Inhibitor Palbociclib in ER-Positive Endocrine-Resistant Breast Cancer. Cancer Res (2018) 78:P4–03. doi: 10.1158/1538-7445.SABCS17-P4-03-05

[101] Martínez-Zamudio RI Roux P-F de Freitas JANLF Robinson L Doré G Sun B. AP-1 Imprints a Reversible Transcriptional Programme of Senescent Cells. Nat Cell Biol (2020) 22:842–55. doi: 10.1038/s41556-020-0529-5 PMC7899185 32514071

[102] Bi M Zhang Z Jiang Y-Z Xue P Wang H Lai Z. Enhancer Reprogramming Driven by High-Order Assemblies of Transcription Factors Promotes Phenotypic Plasticity and Breast Cancer Endocrine Resistance. Nat Cell Biol (2020) 22:701–15. doi: 10.1038/s41556-020-0514-z PMC7737911 32424275

